# How about the evidence assessment tools used in education and management systematic reviews?

**DOI:** 10.3389/fmed.2023.1160289

**Published:** 2023-05-09

**Authors:** Hui Lan, Xuan Yu, Zhe Wang, Ping Wang, Yajia Sun, Zijun Wang, Renfeng Su, Ling Wang, Junxian Zhao, Yue Hu, Shouyuan Wu, Mengjuan Ren, Kehu Yang, Xingrong Liu, Yaolong Chen

**Affiliations:** ^1^School of Public Health, Lanzhou University, Lanzhou, China; ^2^School of Basic Medicine, Lanzhou University, Lanzhou, China; ^3^School of Nursing, Lanzhou University, Lanzhou, China; ^4^Key Laboratory of Evidence Based Medicine and Knowledge Translation of Gansu Province, Lanzhou, China; ^5^Lanzhou University Institute of Health Data Science, Lanzhou, China; ^6^WHO Collaborating Center for Guideline Implementation and Knowledge Translation, Lanzhou, China; ^7^Research Unit of Evidence-Based Evaluation and Guidelines, Chinese Academy of Medical Sciences, Lanzhou, China

**Keywords:** bias, education, evidence based medicine, systematic review, social science

## Abstract

**Objectives:**

To systematically analyze the use of evidence assessment tools in systematic reviews of management and education.

**Study design and setting:**

We systematically searched selected literature databases and websites to identify systematic reviews on management and education. We extracted general information of the included studies and information about the evidence assessment tool they applied, including whether it was used for methodological quality assessment, reporting quality assessment or evidence grading, as well as the name, reference, publication year, version and original intended use of the tool, the role of the tool in the systematic review, and whether the quality determination criteria were given.

**Results:**

A total of 299 systematic reviews were included, of which only 34.8% used evidence assessment tools. A total of 66 different evidence assessment tools were used, of which Risk of Bias (ROB) and its updated version (*n* = 16, 15.4%) were the most frequent. The specific roles of the evidence assessment tools were reported clearly in 57 reviews, and 27 reviews used two tools.

**Conclusion:**

Evidence assessment tools were seldom used in systematic reviews in social sciences. The understanding and reporting of evidence assessment tools among the researchers and users still needs improvement.

## 1. Introduction

Systematic reviews and meta-analyses include evidence based on the inclusion and exclusion criteria to answer a research question, and use methods to reduce bias and random error to draw precise, powerful and convincing conclusions by critically synthesizing evidence that often consists of high-quality randomized controlled trials (1). High quality systematic reviews can provide evidence for decisions made by doctors, researchers, users, and decision-makers (2). Systematic reviews should follow a rigorous methodology to identify and reduce any bias that may affect the interpretation of the results (3). Assessing the methodological quality of the included studies can help to judge the confidence in the results and reduce random and systematic errors (4, 5). Though it is not necessary, assessment of the reporting quality of the included studies can also help researchers and users understand the original studies and determine whether these studies match the objective of the systematic review (6). In addition to the quality assessment of the included studies, evidence grading tools are needed to evaluate the quality of the body of evidence, interpret the results and reduce the risks of bias drawing misleading conclusions from the findings (7). The above three types of tools are used in the process of conducting a systematic review to assess the quality of the included studies and the body of evidence. Therefore, from now on we refer to methodological quality assessment tools, reporting quality assessment tools and grading tools as evidence assessment tools.

Systematic reviews are commonly conducted in health sciences, and conducting a quality assessment has been standard practice since the establishment of evidence-based medicine (8). A systematic review published in 2021 find that 51.6% of systematic reviews of *in vitro* studies used evidence assessment tools, involving 51 different tools (9). Hitherto, Equator Network^1^ has collected over 500 reporting guidelines in health sciences. A study conducted in 2018 analyzed the grading tools recommended by more than 30 guideline development handbooks internationally, and found that 17 different evidence grading tools were used (10). Evidence assessment tools have thus the potential to facilitate research and policymaking, not only in health sciences, but also in other fields of science, as evidence-based medicine is transforming into evidence-based science (11, 12).

Systematic reviews are being increasingly applied in the field of social sciences. The establishment of the Campbell Collaboration Network in 2000 further promotes the development of systematic reviews in social sciences (13), especially in the fields of management and education. Management and education have also been the main topics of systematic reviews conducted in China in social sciences (14). However, the quality of systematic reviews in social sciences in China was concerning, especially the methodological quality, with 87.5% of systematic reviews not assessing the quality of the included studies (14). In fact, the quality of most original studies in social sciences also need to be improved, and the inclusion of studies without an assessment of their quality influences the reliability of the systematic review’s results (15).

The current status of use of evidence assessment tools in systematic reviews in social sciences, including on the most common topics, management and education, is unclear. Thus, a cross-sectional analysis used to perform a systematic analysis of the current state of evidence assessment tools used in published, peer-reviewed systematic reviews on management and education as example of social sciences, and thus provide suggestions for improvement of the quality of systematic reviews in social sciences.

## 2. Materials and methods

### 2.1. Search strategy

This study systematically searched four databases and two websites, EBSCOhost (which includes the Teacher Reference Center, Business Source Premier, The Belt and Road Initiative Reference Source, and ERIC), Web of Science, MEDLINE (via via PubMed), China National Knowledge of Infrastructure (CNKI), Campbell Systematic Reviews, and the International Initiative for Impact Evaluation (3ie), from July 1, 2021 to December 31, 2021. The search terms were composed of the following: systematic review, meta-analysis, meta analysis, education, educate, management, and manage. The exact search strategy is shown in the Supplementary file.

### 2.2. Selection criteria

Systematic reviews published in Chinese or English where the research topic was management or education were included (we included studies that results mainly related to management or education rather than health outcomes). We included all types of systematic reviews, both qualitative and quantitative. We defined systematic review as a study that conducted a systematic search (at least two databases) and literature screening based on inclusion/exclusion criteria to resolve a certain research question.

The following articles were excluded: (1) articles where the full text was not available, (2) duplicates, and (3) meta-analyses without a systematic search.

### 2.3. Study selection and data extraction

After de-duplication with EndNote X9,^2^ two investigators (Hui Lan and Ping Wang) independently screened the literature by reading first the titles and abstracts, and then the full texts of potentially relevant articles. Any disagreements were resolved by discussion or consulting a third investigator (Xuan Yu). We extracted information only from studies that used evidence assessment tools. Seven investigators independently performed the extraction (Hui Lan, Yajia Sun, Ping Wang, Renfeng Su, Ling Wang, Junxian Zhao, and Yue Hu). Any disagreements were resolved by discussion or consulting another investigator (Xuan Yu). We extracted the following information: (1) General information: title, first author, country of the first author, language of publication, study topic (according to the National Standard for Classification and Coding of Disciplines of the People’s Republic of China (GB/T 13745–2009), type of the included original study, whether a meta-analysis was performed, and the article type in the journal; (2) Information about the evidence assessment tool: whether it was used for methodological quality assessment, reporting quality assessment and/or evidence grading, name and reference of the tool, year of publication of the tool, the original intended use of the tool (according to the tool’s reference), the role of the tool in the systematic review (according to the description in the systematic review), version of the tool (original version, extended/updated version or customized version), and whether the quality determination criteria were given.

### 2.4. Data analysis

Microsoft Excel 2019 was used to develop the database for the final included literature. The results were presented descriptively, with absolute numbers and percentages. Statistical significance levels of constituent ratios between reviews on education and on management were calculated using SPSS software (version 25.0).^3^

## 3. Results

### 3.1. Search results

**Figure 1:**
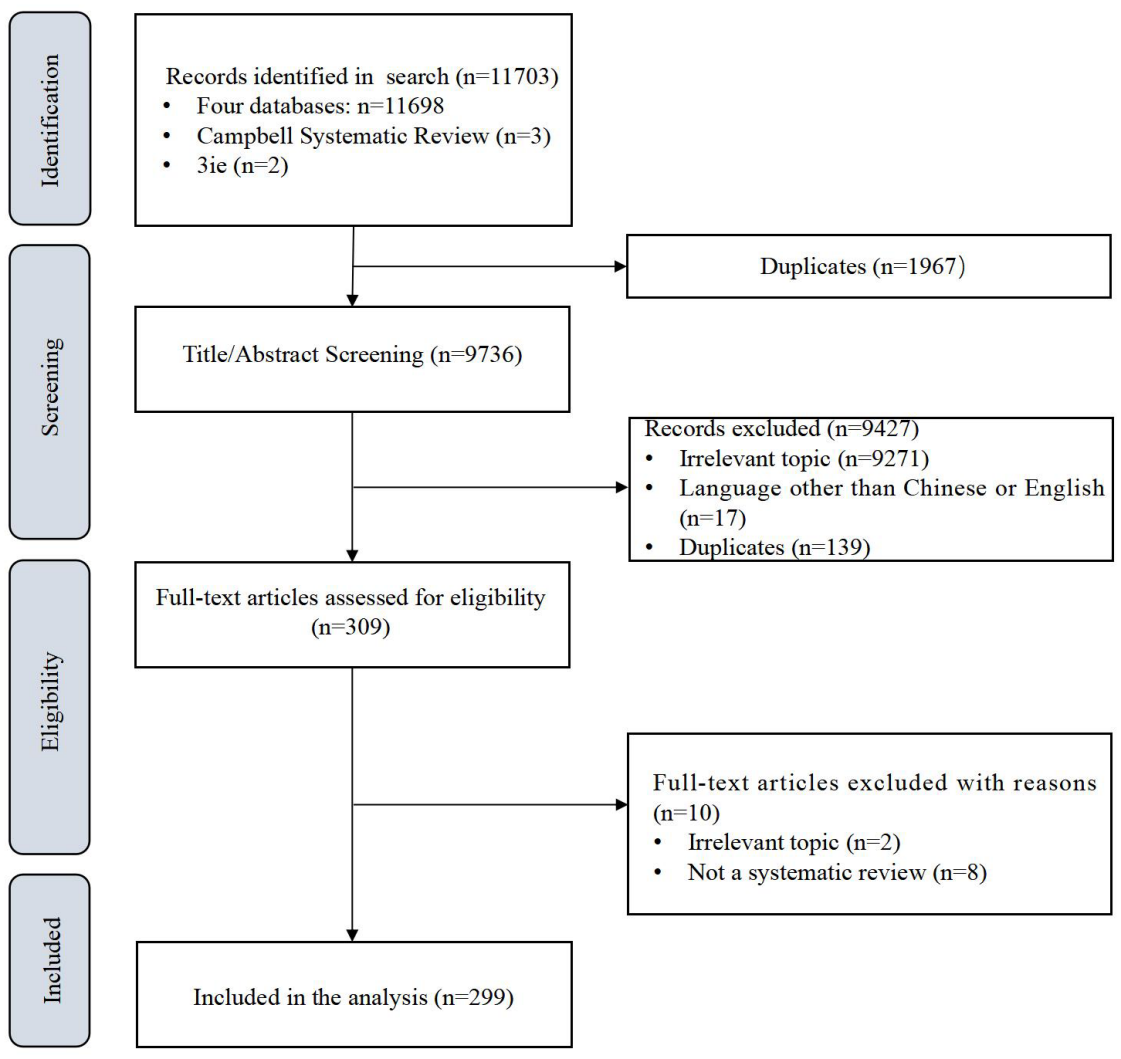
Flow chart of the literature search.

We retrieved 11,703 records from the four electronic databases and two websites and removed 1967 duplicates automatically. After two phases of screening, we finally included 299 reviews. The screening flow is shown in Figure 1 which refer to the preferred reporting items for systematic reviews and meta-analyses (PRISMA) (16).

### 3.2. Basic characteristics of the included studies

Of the 299 systematic reviews, 220 (220/299, 73.6%) focused on education, and 79 (79/299, 26.4%) on management. Only 104 (104/299, 34.8%) reviews used evidence assessment tools. The rate of using evidence assessment tools was higher in the field of education (91/220, 41.4%) than management (13/79, 16.5%) (41.4% vs. 16.5%, *p* < 0.001).

A further analysis of the 104 systematic reviews using evidence assessment tools showed that 91 (91/104, 87.5%) reviews were related to education: 38 (38/91, 41.8%) to higher education, and 11 (11/91, 12.1%) to physical education. Thirteen (13/104, 12.5%) reviews were related to management: four of them focused on “other disciplines in management” (4/13, 30.8%), followed by marketing management (3/13, 23.1%) and health management (3/13, 23.1%). Of the 104 reviews, 94 (94/104, 90.4%) were published in English and 10 (10/104, 9.6%) in Chinese. The first authors came from 25 countries, among which the highest numbers were from United States (18/104, 17.3%), followed by the China (16/104, 15.4%) and the United Kingdom (14/104, 13.5%). Seventy-two (72/104, 69.2%) of the reviews used meta-analysis for data synthesis. Fifty-one (51/104, 49.0%) reviews clearly stated the criteria for evidence assessment, and 53 (53/104, 51.0%) reviews did not mention the assessment criteria. The types of original studies included in the systematic reviews were diverse: the maximum number different study types included in a single systematic review was 30. The details of the characteristics of the included studies are presented in Table 1.

**Table 1:** Characteristics of the included systematic reviews (*n* = 104).

Characteristic		Category	*n* (%)
Study topic^a^	Education		91 (87.5%)
		Higher education	38 (41.8%)
		Physical education	11 (12.1%)
		General education	10 (11.0%)
		Educational technology	9 (9.9%)
		Preschool education	6 (6.6%)
		Educational psychology	6 (6.6%)
		Special education	6 (6.6%)
		Health education	1 (1.1%)
		Other disciplines in education	4 (4.4%)
	Management		13 (12.5%)
		Marketing management	3 (23.1%)
		Health management	3 (23.1%)
		Administration	1 (7.7%)
		Equipment management	1 (7.7%)
		Other disciplines in management	4 (30.8%)
		Other disciplines in business management	1 (7.7%)
Language of publication		Chinese	10 (9.6%)
		English	94 (90.4%)
First author’s country		United States	18 (17.3%)
		China	16 (15.4%)
		United Kingdom	14 (13.5%)
		Australia	12 (11.5%)
		Spain	8 (7.7%)
		Netherlands	4 (3.8%)
		Ireland	3 (2.9%)
		Brazil	3 (2.9%)
		Turkey	3 (2.9%)
		Germany	2 (1.9%)
		Finland	2 (1.9%)
		South Korea	2 (1.9%)
		Switzerland	2 (1.9%)
		Singapore	2 (1.9%)
		Iran	2 (1.9%)
		Denmark	2 (1.9%)
		Canada	2 (1.9%)
		Malaysia	1 (1.0%)
		Norway	1 (1.0%)
		Portugal	1 (1.0%)
		Saudi Arabia	1 (1.0%)
		Chile	1 (1.0%)
		New Zealand	1 (1.0%)
		India	1 (1.0%)
Number of types of original studies included in the review^b^		1 ~ 5	60 (57.7%)
		6 ~ 10	8 (7.7%)
		20	1 (1.0%)
		30	1 (1.0%)
		Impossible to determine	36 (34.6%)
Meta-analysis conducted		Yes	72 (69.2%)
		No	32 (30.8%)
Explanation of the evidence assessment criteria		Yes	51 (49.0%)
		No	53 (51.0%)

a Study topic was defined according to the National Standard for Classification and Coding of Disciplines of the People’s Republic of China (GB/T 13745–2009).

b We used each review’s own categorization to determine the study types among the included studies.

### 3.3. Evidence assessment tools used in systematic reviews

**Figure 2:**
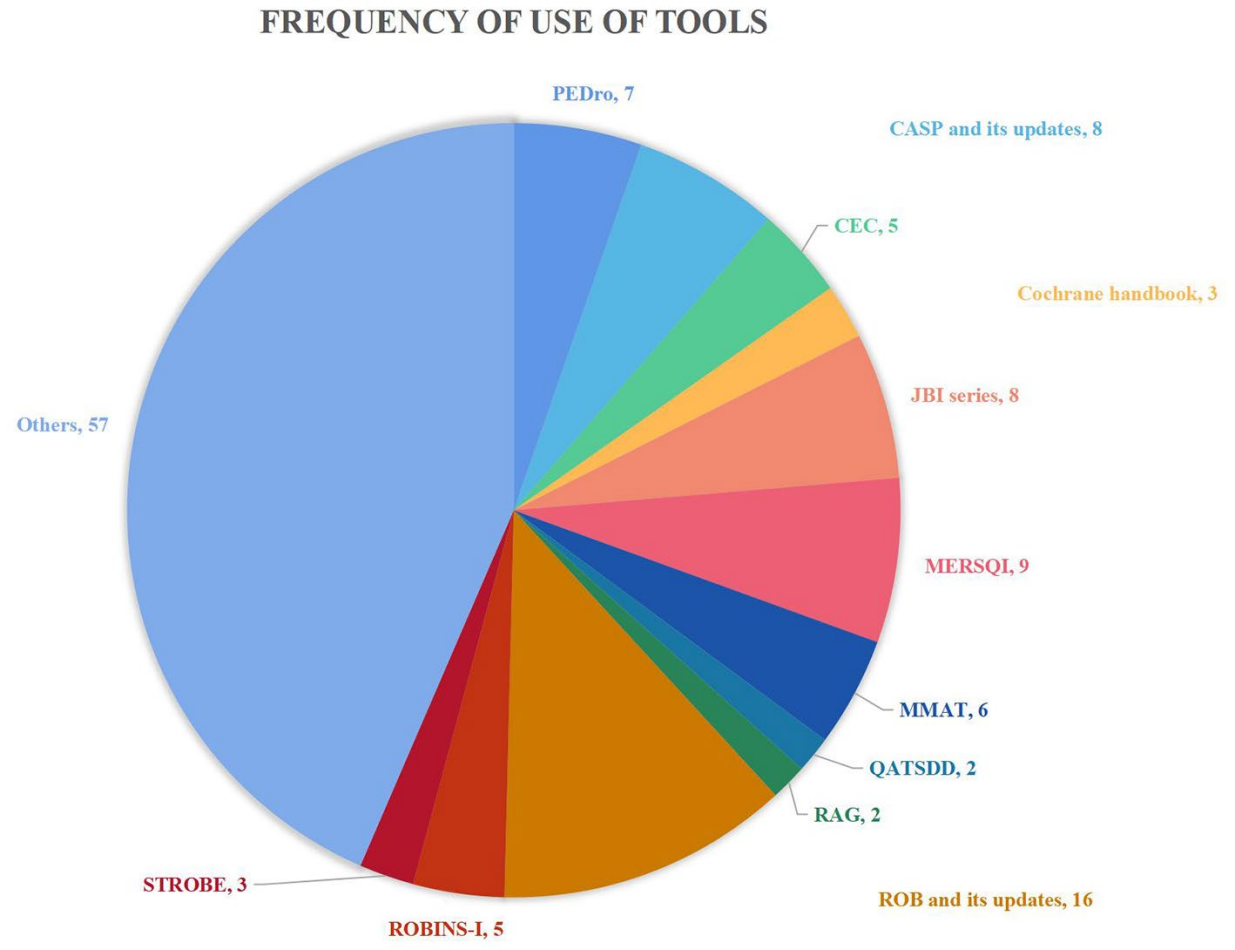
The number of systematic reviews using each evidence assessment tool.

We identified a total of 66 assessment tools from the 104 systematic reviews. The most frequently used tool was the Risk of Bias (ROB) and its updated version (16/104, 15.4%), followed by the Medical Education Research Quality Instrument (MERSQI) (9/104, 8.7%). The frequency of tool usage is shown in Figure 2. Twenty-seven reviews used two different tools, ten of which did not report the specific roles of the tool, and 17 studies reported using tools for methodological quality assessment (see section “Methodological quality assessment tools”). Of the 66 evidence assessment tools (Table 2; Supplementary file), 63 were used for quality assessment (methodological or reporting quality) and three for evidence grading. Forty-eight tools were used according to the references of the tools, 11 tools were adapted and used by authors of systematic reviews and seven tools were created by the authors of systematic reviews. Systematic reviews on management used a total of 14 different tools, and systematic reviews on education 57 tools. Five tools were applied in systematic reviews in both fields (management and education): ROB, The Joanna Briggs Institute (JBI) tool, Mixed Methods Appraisal Tool (MMAT), Strengthening the Reporting of Observational Studies in Epidemiology (STROBE), and Consolidated Standards of Reporting Trials (CONSORT). Systematic reviews published in Chinese used six and reviews published in English 62 different tools, ROB and Cochrane Handbook being used by reviews in both languages.

**Table 2:** Characteristics of the evidence assessment tools and their use in the systematic reviews.

Characteristic	Category	*n* (%)
The role of tools reported in systematic reviews (*N* = 104)	The use of the tool explicitly reported:	57 (54.8%)
	Methodological quality assessment*	55 (96.5%)
	Reporting quality assessment*	4 (7.0%)
	Evidence grading*	3 (5.3%)
	The use of evidence assessment tools not explicitly reported (only as quality assessment)	47 (45.2%)
Number of quality assessment tools used in the systematic review (*N* = 104)	1	77 (74.0%)
	2	27 (26.0%)
Version of the tool (*N* = 66)	Original version	48 (72.7%)
	Adapted version	11 (16.7%)
	Custom version	7 (10.6%)
Number of tools applied to each research topic (*N* = 66)	Management	14 (21.2%)
	Education	57 (86.4%)
Languages of the systematic reviews using each tool (*N* = 66)	Chinese	6 (9.1%)
	English	62 (93.9%)
Original intended use of the tool (*N* = 66)	Quality assessment	63 (95.5%)
	Evidence grading	3 (4.5%)

*Two studies reported to use the tool for both methodological quality assessment and reporting quality assessment. Two studies reported to use the tool for both methodological quality assessment and evidence grading.

The analysis of the 57 (57/104, 54.8%) studies that explicitly reported the specific use of the assessment tool found that 55 studies reported to use the tool for methodological quality assessment, four studies for reporting quality assessment, and three studies for evidence grading. Meanwhile, no study explicitly reported using the assessment tools for all three purposes. The reported use of the tool in most of the studies (51/57, 89.5%) was consistent with the use mentioned in the tool’s instructions.

#### 3.3.1. Methodological quality assessment tools

Fifty-five reviews reported explicitly to have used the evidence assessment tools for methodological quality assessment. Forty-nine (49/55, 89.1%) studies used tools specifically intended for the assessment of methodological quality, and for six (6/55, 10.9%) studies it was not possible to determine the original intended use of the tool because the references were not available. Seventeen (17/55, 30.9%) reviews explicitly reported using two tools: 12 (12/55, 21.8%) reviews used two tools for methodological quality assessment, three (3/55, 5.5%) reviews used one tool for methodological quality assessment and one for reporting quality assessment, and two (2/55, 3.6%) reviews one tool for methodological quality assessment and one for evidence grading. Fifty-three (53/55, 96.4%) of the reviews were on education, and two (2/55, 3.6%) on management. The ROB tool was the only methodological quality assessment tool used for systematic reviews in both fields, management and education. Four of the 55 studies were published in Chinese and 51 in English. The ROB tool was the only methodological quality assessment tool used by both Chinese- and English-language reviews. The systematic reviews reported a total of 34 different tools for methodological quality assessment, with details shown in the Supplementary file.

#### 3.3.2. Reporting quality assessment tools

Four reviews explicitly reported using reporting quality assessment tools. Four reviews used tools specifically intended for reporting quality assessment; one review did not provide reference for the tool so we could not judge its original intended use. Three of the reviews were on education and one on management, and all four reviews were published in English. The four reviews used three different quality assessment tools including QRT (Quality Rating Tool, without reference), RAG, and CONSORT (Supplementary file).

#### 3.3.3. Evidence grading tools

Three reviews explicitly reported using assessment tools for evidence grading, all being tools specifically intended for evidence grading. All three reviews were in the field of education and published in English. All three reviews used different tools GRADE (Grading of Recommendations Assessment, Development and Evaluation), GRADE-CERQual (Grading of Recommendations Assessment, Development and Evaluation- Confidence in the Evidence from Reviews of Qualitative Research), American Speech-Language-Hearing Association^,^ technical report (Supplementary file).

## 4. Discussion

Only one third of the systematic reviews about management and education used quality assessment or evidence grading tools to assess the quality of the included studies or to grade the body of evidence. Among the reviews, a total of 66 different tools were used. None of the studies evaluated all three aspects: methodological quality, reporting quality and the grade of the evidence.

We found that systematic reviews in the field of social sciences reported the use of the tools poorly. This is consistent with the results of a study published in 2021 (14): the reporting quality of systematic reviews in the field of social sciences was concerning and reporting was not fully standardized. In addition, we further analyzed the consistency between the reported use and the intended use of the tools. Nearly half of the studies did not explicitly report the way the tools were used, and it was therefore often impossible to further judge whether they were used correctly. The interdisciplinary use of evidence assessment tools brings challenges to researchers. Therefore, there is a need to strengthen the knowledge and attention on quality assessment among researchers in the field of social sciences and develop quality assessment tools applicable to social sciences.

We found four tools applied in reviews on both management and education, and two tools applied in both Chinese- and English-language studies. The ROB tool was included in both of these groups. Since the concept of “randomization” was introduced in 1925 in the field of agriculture, randomized controlled trials (RCT) have become gradually more common also in the field of social sciences (17–19). A study published in 2022 found that the number of RCTs in social sciences published every year rose rapidly between 2000 to 2020 (15). RCT is generally considered to be one of the strongest study designs (20). The ROB tool, a methodological quality assessment tool for RCTs (21), has been continuously improved since it was originally developed in 1961, and adapted versions for other study types have been derived. Of the many existing adaptations of ROB, the most commonly used one is the Cochrane Collaboration’s tool for assessing the risk of bias in randomized trials (ROB 1.0), which was published in 2008 and updated in 2011. Thus, ROB is also available for use in RCTs of social sciences to assess and improve methodological quality. Other methodological quality assessment tools for different types of research including NOS (Newcastle-Ottawa Scale), JBI, CASP (The Critical Appraisal Skills Programme), et al.

Compared with systematic reviews in health sciences (especially in medicine), the use of evidence assessment tools in social sciences is less common. Only a few studies assessed the reporting quality or graded the body of evidence. In 2016, a study on the use of evidence assessment tools in systematic reviews on medical research found that 71.8% of them assessed the evidence with a total of 51 different tools, most of which were for the assessment of methodological quality (22). Although systematic review as a research method is increasingly gaining attention and being promoted and applied in social sciences, the quality of the systematic reviews was below the average level (14). May related to they have not paid enough attention to the use of evidence assessment tools when producing systematic reviews.

The evidence assessment tools used in the field of social sciences were found to be partly the same as those used in the field of medicine. We found ROB, JBI, NOS, STROBE, GRADE and GRADE-CERQual to be commonly used. At present, evidence assessment tools tailored for the field of social sciences are still lacking. Researchers may use evidence assessment tools from the field of medicine (23). However, some items of the evidence assessment tools developed for medical research may not be fully applicable to assess articles in other fields of research such as social sciences. Furthermore, the evidence assessment tools used in some studies were adapted from the exiting tools in the field of medicine or defined by the researchers themselves without being published or used before. Therefore, researchers need to carefully consider the results of the assessment due to the differences in research types between social sciences and health sciences.

We give the following four suggestions for future research based on the findings of this study: (1) More attention needs to be paid on the application of the tools to assess the quality of original research and body of evidence, and methodologists in evidence-based science should be invited to review the results of the assessment; (2) Evidence assessment tools adapted from health sciences must be used correctly to ensure their applicability in social sciences and other fields; (3) Assessment tools that are suitable for different research types in the field of social sciences need to be developed; and (4) The standardization and transparency of systematic reviews need improvement: the quality of systematic reviews in social sciences could be further enhanced through more training for researchers in the field.

Our study has several limitations. First, we did not report the correspondence between study types and different tools when a systematic review used two different tools or included multiple types of original research. Therefore, we did not attempt to evaluate the correctness of the use of each tool. Second, this study only focused on the systematic reviews published within a time period of six months, but we believe that thanks to the large sample size the results are at least to some extent representative of the overall situation. Third, we searched several databases systematically, but did not conduct a manual search of the most relevant journals in the field. Although the searched databases may include these key journals, we may have missed some high-quality systematic reviews. Fourthly, due to the fact that the definition of systematic review may not be well known to systematic review researchers, some of the systematic reviews included in this study may actually be scoping reviews or evidence mapping, and therefore the results of this study may be underestimated.

## 5. Conclusion

In conclusion, the proportion of systematic reviews and meta-analyses in the field of social sciences (management and education) that assessed the quality of the evidence is low. Although some systematic reviews in social sciences adapted tools from health sciences to assess the evidence, most of them did not report the exact way the tool was used. Therefore, researchers in social sciences need to improve the understanding and reporting of the utilization of evidence assessment tools.

## Data availability statement

The original contributions presented in the study are included in the article/supplementary material, further inquiries can be directed to the corresponding authors.

## Ethics statement

This manuscript is a cross-sectional analysis of a review article and does not involve a research protocol requiring approval by the relevant institutional review board or ethics committee.

## Author contributions

HL: literature search, screening, data extraction, statistical analysis, wrote the draft, and the full text. XY: data extraction, draft modification, and study design. ZW: study design and manuscript modification. PW: literature screening, data extraction, and draft modification. YS: data extraction and draft modification. RS, LW, ZW, and YH: data extraction. SW and MR: methodology support. KY: study design, methodology support, and supervision. XL: study design, methodology support, supervision, and manuscript modification. YC: study design, methodology support, supervision, manuscript modification, and funding acquisition. All authors contributed to the article and approved the submitted version.

## Funding

This work was supported by the National Social Science Foundation of China: “Research on the Theoretical System, International Experience and Chinese Path of Evidence-based Social Science” (Project No. 19ZDA142). The funding source had no role in design of study, the collection, analysis, interpretation, and the decision to approve publication of the finished manuscript. The authors are grateful to Janne Estill (Institute of Global Health, University of Geneva, Geneva, Switzerland) for the modification of the full text.

## Conflict of interest

The authors declare that the research was conducted in the absence of any commercial or financial relationships that could be construed as a potential conflict of interest.

## Publisher’s note

All claims expressed in this article are solely those of the authors and do not necessarily represent those of their affiliated organizations, or those of the publisher, the editors and the reviewers. Any product that may be evaluated in this article, or claim that may be made by its manufacturer, is not guaranteed or endorsed by the publisher.

## Supplementary material

The Supplementary material for this article can be found online at: https://www.frontiersin.org/articles/10.3389/fmed.2023.1160289/full#supplementary-material

## Footnotes

^1^https://www.equator-network.org/

^2^https://endnote.com/

^3^https://www.ibm.com/products/spss-statistics

## Abbreviations

PEDro: Physiotherapy Evidence Database scale
CASP: The Critical Appraisal Skills Programme
CEC: Council for Exceptional Children
JBI: The Joanna Briggs Institute
MERSQI: The Medical Education Research Quality Instrument
MMAT: The Mixed Methods Appraisal Tool
QATSDD: The Quality Assessment Tool for Studies with Diverse Designs
RAG: Red-Amber-Green
ROB: Risk of Bias
ROBINS-I: The Risk of Bias in Non-Randomized Studies of Interventions
STROBE: Strengthening the Reporting of Observational Studies in Epidemiology
NOS: Newcastle-Ottawa Scale
GEADE: Grading of Recommendations Assessment, Development and Evaluation
CONSORT: Consolidated Standards of Reporting Trials
GRADE-CERQual: Grading of Recommendations Assessment, Development and Evaluation- Confidence in the Evidence from Reviews of Qualitative Research
