# Supplementary material for: How about the evidence assessment tools used in education and management systematic reviews?
Source: Front Med (Lausanne). 2023 May 9;10:1160289. doi: 10.3389/fmed.2023.1160289 (PMC10203209; doi:10.3389/fmed.2023.1160289)
Supplement: Supplementary file 1 [file Data_Sheet_1.docx]

**Search Strategy**

**PubMed（n=6821）**

1. "systematic review"[Mesh]
2. "meta-analysis"[Mesh]
3. "systematic review"[title]
4. "meta-analysis"[title]
5. "meta analysis"[title]
6. OR #1-#5
7. "education"[Mesh]
8. "education"[all fields]
9. "educate"[all fields]
10. "manage"[all fields]
11. "management"[all fields]
12. OR #7-#11
13. July 2021 - December 2021
14. #6 AND #12 AND #13

**EBSCOhost（n=615）**

1. "systematic review"[TI]
2. "meta-analysis"[TI]
3. "meta analysis"[TI]
4. OR #1-#3
5. "education"[TX]
6. "educate"[TX]
7. "management"[TX]
8. "manage"[TX]
9. OR #5-#8
10. July 2021 - December 2021
11. #4 AND #9 AND #10

**WOS（n=4220）**

#1. "systematic review"[TITLE]

#2. "meta-analysis"[TITLE]

#3. "meta analysis"[TITLE]

#4. OR #1-#3

#5. "management"[TOPIC]

#6. "manage"[TOPIC]

#7. "education"[TOPIC]

#8. "educate"[TOPIC]

#9. OR #5-#8

#10. July 2021 - December 2021

#11. #4 AND #9 AND #10

**Campbell Systematic Reviews (n=3)**

**International Initiative for Impact Evaluation (n=2)**

**CNKI（n=42）**

#1. 系统评价[篇名]

#2. 系统综述[篇名]

#3. Meta分析[篇名]

#4. Meta-分析[篇名]

#5. 荟萃分析[篇名]

#6. 元分析[篇名]

#7. OR #1-#6

#8. 教育[主题]

#9. 教育学[主题]

#10. 管理[主题]

#11. 管理学[主题]

#12. OR #8-#11

#13. 2021年7月-2021年12月

#14. #7 AND #12 AND #13
