# Supplementary material for: How about the evidence assessment tools used in education and management systematic reviews?
Source: Front Med (Lausanne). 2023 May 9;10:1160289. doi: 10.3389/fmed.2023.1160289 (PMC10203209; doi:10.3389/fmed.2023.1160289)
Supplement: Supplementary file 2 [file Data_Sheet_2.docx]

**Tool Details**

| **Name or acronym** | **Use of the tool** | **References (description/introduction of the tool)** | **Study types included in the systematic reviews** | **Number of systematic reviews using the tool （n, %）** |
| --- | --- | --- | --- | --- |
| ROB (Randomized Controlled Trial) and its updates | Methodological quality | Lundh A, Gøtzsche P. Recommendations by Cochrane Review Groups for assessment of the risk of bias in studies. BMC Med Res Methodol. 2008 Apr 21;8:22.  Higgins , Altman DG, Gøtzsche P, et al. The Cochrane Collaboration's tool for assessing risk of bias in randomised trials. BMJ. 2011 Oct 18;343:d5928.  Sterne J, Savović J, Page M, et al. RoB 2: a revised tool for assessing risk of bias in randomised trials. BMJ. 2019 Aug 28;366:l4898.  Ma L, Wang Y, Yang Z, et al. Methodological quality (risk of bias) assessment tools for primary and secondary medical studies: what are they and which is better? Mil Med Res. 2020 Feb 29;7(1):7.  Higgins J, Green S. Cochrane handbook for systematic reviews of interventions:cochrane book series. Chichester: Wiley, 2008.. | single-case design study, group design study, non-equivalent control study, single group pre- and post-intervention study, RCT (Randomized Controlled Trial), observational study, non‐randomized study, cross-sectional study, longitudinal study, quantitative study, qualitative study, mixed design study, quasi experimental study, controlled trial, pre-/post-test, randomized comparative study, pre-/post-session self-assessment, longitudinal survey study, observational cohort, evaluation of self-reported questionnaire after teaching, pre-/post-teaching questionnaire, evaluation of self-reported questionnaires before and after, pilot study, focus group study, evaluation of survey conducted after each workshop, post-hoc interviews | 16, 15.4% |
| MERSQI (The Medical Education Research Quality Instrument) | Methodological quality | Reed D, Cook D, Beckman T, et al Association between funding and quality of published medical education research. JAMA. 2007 Sep 5;298(9):1002-9.  Reed D, Beckman T, Wright S, et al. Predictive validity evidence for medical education research study quality instrument scores: quality of submissions to JGIM's Medical Education Special Issue. J Gen Intern Med. 2008 Jul;23(7):903-7.  Cook D, Reed D. Appraising the quality of medical education research methods: the Medical Education Research Study Quality Instrument and the Newcastle-Ottawa Scale-Education. Acad Med. 2015 Aug;90(8):1067-76. . | cohort study, RCT, pre- and post- test evaluation, controlled study, randomized trial, cluster randomized trial, time-series design study, before‐after study, preoperative and postoperative study, pilot study, letters, brief reports/innovations, articles/commentaries, cross-sectional study, quantitative descriptive study, mixed-methods study, quasi experimental study, proof-of-concept trial | 9, 8.7% |
| CASP (The Critical Appraisal Skills Programme) and its updates | Methodological quality | Zeng X, Liu H, Chen X, et al. Meta-analysis series 4: Quality evaluation tools for observational study. Chinese Journal of evidence based cardiovascular medicine. 2012,4(04):297-299.  Critical Appraisal Skills Programme . Making sense of evidence about clinical effectiveness. 10 questions to help you make sense of qualitative research. Oxford: CASP; 2018.  Critical Appraisal Skills Programme . CASP Checklist: 12 questions to help you make sense of a Cohort Study. Oxford: CASP; 2018. | randomized trial, grounded theory, focus group discussion, semi-structured interviews, open questionnaire evaluation, quantitative study, evaluation before and after control, focus group study, survey, cross-sectional study, quasi experimental study, pre- and post-test evaluation, post test evaluation, cohort study, intervention study, observational study, descriptive study, case series, phenomenological study, case study, ground theory, narrative inquiry, participatory study | 8, 7.7% |
| JBI (The Joanna Briggs Institute) | Methodological quality | Dixon-Woods M, Sutton A, Shaw R, et al. Appraising qualitative research for inclusion in systematic reviews: a quantitative and qualitative comparison of three methods. J Health Serv Res Policy. 2007 Jan;12(1):42-7.  The Joanna Briggs Institute Critical Appraisal tools for use in JBI Systematic Reviews. Checklist for Quasi-Experimental Studies (non-randomized experimental studies). 2017.  The Joanna Briggs Institute Critical Appraisal tools for use in JBI Systematic Reviews. Checklist for Randomized Controlled Trials. 2017. | non-randomized trial, cohort study, pre- and post-test quasi experimental study, mixed methods research (theme analysis）, qualitative study, grounded theory, constant comparative analysis, interpretive phenomenological analysis, basic interpretive approach, quasi experimental study, RCT, crossover trial, comparative study, | 8, 7.7% |
| PEDro (Physiotherapy Evidence Database ) | Methodological quality | Maher C, Sherrington C, Herbert R, et al. Reliability of the PEDro scale for rating quality of randomized controlled trials. Phys Ther. 2003 Aug;83(8):713-21.  Elkins M, Moseley A, Sherrington C, et al. Growth in the Physiotherapy Evidence Database (PEDro) and use of the PEDro scale. Br J Sports Med. 2013 Mar;47(4):188-9. | RCT, quasi-experimental designs, pre-post intervention testing, descriptive study, interventional study, observational study | 7, 6.7% |
| MMAT (The Mixed Methods Appraisal Tool) | Methodological quality | Pace R, Pluye P, Bartlett G, et al. Testing the reliability and efficiency of the pilot Mixed Methods Appraisal Tool (MMAT) for systematic mixed studies review. Int J Nurs Stud. 2012 Jan;49(1):47-53.  Hong Q, Fàbregues S, Bartlett G, et al. The Mixed Methods Appraisal Tool (MMAT) version 2018 for information professionals and researchers. Education for Information. 2018; 34(4): 285-291.  Hong Q, Pluye P, Fàbregues S, et al. Improving the content validity of the mixed methods appraisal tool: a modified e-Delphi study. J Clin Epidemiol. 2019 Jul;111:49-59.e1. | cross-sectional study, randomized trial, cluster randomized trial, time-series design study, pre-post control study, preoperative-postoperative study, pilot study, quasi experimental pre-test and post-test single group study, descriptive study, mixed method study, qualitative study, RCT, non-randomized study, quantitative descriptive study | 6, 5.8% |
| ROBINS-I (The Risk of Bias in Non-Randomized Studies of Interventions) | Methodological quality | Sterne J, Hernán M, Reeves B, et al. ROBINS-I: a tool for assessing risk of bias in non-randomised studies of interventions. BMJ. 2016 Oct 12;355:i4919. | RCT, non-randomized trial, non-equivalent control study, single group pre- and post-intervention study, evaluation before and after control, focus group study, survey, descriptive study, quasi experimental study, pre- and post-trial evaluation, post test evaluation | 5, 4.8% |
| CEC (Council for Exceptional Children) | Methodological quality | Cook B, Buysse V, Klingner J, et al. Council for Exceptional Children: Standards for evidence-based practices in special education. Teaching Exceptional Children, 2014, 46(6): 206.  Cook B, Buysse V, Klingner J, et al. CEC’s standards for classifying the evidence base of practices in special education. Remedial and Special Education, 2015, 36(4): 220-234.. | crossover design study, intervention study, quasi-experimental design study, single-case design study, group design study | 5, 4.8% |
| STROBE (Strengthening the Reporting of Observational Studies in Epidemiology) | Reporting quality | Von Elm E, Altman D, Egger M, et al. The Strengthening the Reporting of Observational Studies in Epidemiology (STROBE) Statement: guidelines for reporting observational studies. Int J Surg. 2014 Dec;12(12):1495-9.  Pouralajal J, Tajik P, Yazdizadeh B, et al. Quality assessment of the reporting of cohort studies before STROBE statement. 2009.  Vandenbroucke J, von Elm E, Altman D, et al. Strengthening the Reporting of Observational Studies in Epidemiology (STROBE): explanation and elaboration. Int J Surg. 2014 Dec;12(12):1500-24. | cross-sectional study, self-report questionnaire evaluation, semi-structured interviews, focus group interviews, evaluation of researchers’ field notes, pre-/post-test evaluation, randomized comparative study, pre-/post-session self-assessment, RCT, longitudinal survey study, observational cohort study, evaluation of pre-/post-teaching questionnaires, survey, randomized comparative study, pilot study, observational study, focus group study, evaluation of surveys after each workshop, post-hoc interviews | 3, 2.9% |
| Cochrane | Methodological quality | Higgins J, Green S. Cochrane handbook for systematic review of intervention V 5.20. 2008. | RCT, quasi-randomized control trial, non-equivalent randomized controlled trial, cluster randomized controlled trial, quasi experimental study | 3, 2.9% |
| QATSDD (The Quality Assessment Tool for Studies with Diverse Designs) | Methodological quality | Sirriyeh R, Lawton R, Gardner P, et al. Reviewing studies with diverse designs: the development and evaluation of a new tool. J Eval Clin Pract. 2012 Aug;18(4):746-52. | RCT, non-randomized intervention study, observational study, qualitative study | 2, 1.9% |
| RAG (Red-Amber-Green) | Reporting quality | Reed D, Cook D, Beckman T, et al. Association between funding and quality of published medical education research. JAMA. 2007 Sep 5;298(9):1002-9.  Gordon M, Gibbs T. STORIES statement: publication standards for healthcare education evidence synthesis. BMC Med. 2014 Sep 3;12:143. | questionnaire investigation, pre-/ post-intervention study, interview, focus group study, observational study, audit, progress report analysis, presentations and reflective journals, report, comment, letter, innovation study | 2, 1.9% |
| CONSORT (Consolidated Standards of Reporting Trials) | Reporting quality | Moher D, Schulz K, Altman D et al. The CONSORT statement: revised recommendations for improving the quality of reports of parallel-group randomized trials. JAMA. 2001 Apr 18;285(15):1987-91. | self-report questionnaire, semi-structured interview, focus group interview, researchers' field notes | 2, 1.9% |
| WWC (What Works Clearinghouse) | Methodological quality | Institute of education sciences. what works clearinghouse standards hand book (Ver. 4.1). 2020. | RCT | 2, 1.9% |
| WOE | / | Gough, David. Weight of Evidence: a framework for the appraisal of the quality and relevance of evidence. Research Papers in Education. 22 (2007): 213 - 228., 22(2), 213–228. | RCT and quasi-experimental | 2, 1.9% |
| Levels of Evidence | Evidence grading | Randall R. Levels of Evidence. ASHA Leader. 2004 | crossover design study, interventional study | 1, 1.0% |
| GRADE (Grading of Recommendations Assessment, Development and Evaluation) | Evidence grading | Balshem H, Helfand M, Schünemann H, et al. GRADE guidelines: 3. Rating the quality of evidence. J Clin Epidemiol. 2011 Apr;64(4):401-6. .. | RCT | 1, 1.0% |
| GRADE-CERQual (Grading of Recommendations Assessment, Development and Evaluation- Confidence in the Evidence from Reviews of Qualitative Research) | Evidence grading | Lewin S, Booth A, Glenton C, et al. Applying GRADE-CERQual to qualitative evidence synthesis findings: introduction to the series. Implement Sci. 2018 Jan 25;13(Suppl 1):2. | descriptive study, multi-case study, phenomenological study, case study, grounded theory, narrative inquiry, participatory study | 1, 1.0% |
| ISPOR (The International Society for Pharmacoeconomics and Outcomes Research checklist) | Quality assessment | / | quantitative study | 1, 1.0% |
| Self-created | Methodological quality | / | cross-sectional study, longitudinal study, experimental study | 1, 1.0% |
| A rating system adapted from Siebes, Wijnroks, and Vermeer | Methodological quality | Siebes R, Wijnroks L, Vermeer A. Qualitative analysis of therapeutic motor intervention programmes for children with cerebral palsy: an update. Dev Med Child Neurol. 2002 Sep;44(9):593-603. | studies other than RCTs | 1, 1.0% |
| AIRE | Methodological quality | Appraisal of indicators through research and evaluation (AIRE). (http://allie.dbcls.jp/cooccur/AIRE;Appraisal+of+Indicators+through+Research+and+Evaluation.html) | literature review, qualitative interview with field visit, workshop, experts’ panel consensus techniques, conceptual map, logical theoretical model, 2-round delphi consensus techniques | 1, 1.0% |
| An adapted version of the Downs and Black instrument | Methodological quality | Downs S, Black N. The feasibility of creating a checklist for the assessment of the methodological quality both of randomised and non-randomised studies of health care interventions. J Epidemiol Community Health. 1998 Jun;52(6):377-84. | cohort study, prospective study, pre- and post-intervention study, cross-sectional study, mixed methods study | 1, 1.0% |
| An adapted version of the OHAT tool | Methodological quality | Rooney A, Boyles A, Wolfe M, et al. Systematic review and evidence integration for literature-based environmental health science assessments. Environ Health Perspect. 2014 Jul;122(7):711-8. | cross-sectional study, retrospective cohort study, longitudinal study | 1, 1.0% |
| Delphi List Verhagen et al | Methodological quality | Verhagen A, De-Vet HC, De-Bie R, et al. The Delphi list: a criteria list for quality assessment of randomized clinical trials for conducting systematic reviews developed by Delphi consensus. J Clin Epidemiol. 1998 Dec;51(12):1235-41. | RCT, non-randomized controlled trial | 1, 1.0% |
| Dixon-Woods et al.’s (2007) criteria | Methodological quality | Lockwood C, Munn Z, Porritt K. Qualitative research synthesis: methodological guidance for systematic reviewers utilizing meta-aggregation. Int J Evid Based Healthc. 2015 Sep;13(3):179-87. | mixed method research (subject analysis), qualitative study, grounded theory, constant comparative analysis, interpretive phenomenological analysis, basic interpretive approach | 1, 1.0% |
| EPHPP (The Effective Public Health Practice Project Quality Assessment Tool) | Methodological quality | Thomas B, Ciliska D, Dobbins M, et al. A process for systematically reviewing the literature: providing the research evidence for public health nursing interventions. Worldviews Evid Based Nurs. 2004;1(3):176-84. | RCT, controlled clinical trial, studies with pre-post design,a control group and acute interventions | 1, 1.0% |
| Instruments developed for single-case experimental methods/group designs | Methodological quality | Romeiser L, Hickman R, Harris S, et al. Single-subject research design: recommendations for levels of evidence and quality rating. Dev Med Child Neurol. 2008 Feb;50(2):99-103.  Downs S, Black N. The feasibility of creating a checklist for the assessment of the methodological quality both of randomised and non-randomised studies of health care interventions. J Epidemiol Community Health. 1998 Jun;52(6):377-84. | case study, supply chain management study, group-comparison analysis | 1, 1.0% |
| NICE (National Institute for Health and Care Excellence) | Methodological quality | National Institute for Health and Care Excellence (2012). Appendix F Quality appraisal checklist – quantitative intervention studies. National Institute for Health and Care Excellence. | two-arm non-randomized intervention study, single-arm intervention study, three-arm randomized intervention study | 1, 1.0% |
| QATQS (Quality Assessment Tool for Quantitative Studies) | Methodological quality | Thomas B, Ciliska D, Dobbins M, et al. A process for systematically reviewing the literature: providing the research evidence for public health nursing interventions. Worldviews Evid Based Nurs. 2004;1(3):176-84. | RCT, quasi-randomized experimental study, pre-post study with a control group, observational study | 1, 1.0% |
| QUADAS-2 (the Quality of Diagnostic Accuracy Studies version 2 checklist) | Methodological quality | Whiting P, Rutjes A, Westwood M, et al. QUADAS-2 Group. QUADAS-2: a revised tool for the quality assessment of diagnostic accuracy studies. Ann Intern Med. 2011 Oct 18;155(8):529-36. | randomized trial, controlled before-after design study, before-after intervention study, exploratory pilot study | 1, 1.0% |
| SURE (Specialist Unit for Review Evidence) | Methodological quality | Specialist Unit for Review Evidence (SURE) (2018). Questions to assist with the critical appraisal of cross-sectional studies. Available at: http://www.cardiff.ac.uk/insrv/libraries/sure/checklists.ht | cross-sectional study | 1, 1.0% |
| the adapted version of the Cochrane Collaboration tool | Methodological quality | Higgins J, Altman D, Gøtzsche P, et al. The Cochrane Collaboration's tool for assessing risk of bias in randomised trials. BMJ. 2011 Oct 18;343:d5928. | cohort study, prospective study, pre- and post-intervention study, cross-sectional study, mixed-methods study | 1, 1.0% |
| The tool developed by Reichow et al. (2018),SCD | Methodological quality | Reichow B, Barton E, Maggin D. Development and applications of the single-case design risk of bias tool for evaluating single-case design research study reports. Res Dev Disabil. 2018 Aug;79:53-64. | single-case design study, group design study | 1, 1.0% |
| Self-created | Methodological quality | Maher C, Sherrington C, Herbert R, et al. Reliability of the PEDro scale for rating quality of randomized controlled trials. Phys Ther. 2003 Aug;83(8):713-21. | study with pre-post design ad a control group | 1, 1.0% |
| An adaptation of the De Lange et al. (2003) evaluation tool. | Methodological quality | De L, Taris T, Kompier M, Houtman I, et al. "The very best of the millennium": longitudinal research and the demand-control-(support) model. J Occup Health Psychol. 2003 Oct;8(4):282-305. | longitudinal study | 1, 1.0% |
| / | Methodological quality | Tufanaru C, Munn Z, Aromataris E, et al. Chapter 3:  Systematic reviews of effectiveness. In: Aromataris E, Munn Z. (Eds.). JBI Manual for Evidence Synthesis. JBI. 2020. | RCT, non-randomized controlled trial, quasi experimental study, pre- post control study, mixed-methods study | 1, 1.0% |
| NOS (The Newcastle-Ottawa Scale) | Methodological quality | Wells G, Shea B, Connell D, et al. The Newcastle-Ottawa Scale (NOS) for Assessing the Quality of Nonrandomised Studies in Meta-Analyses (2021). Available from: http://www.ohri.ca/programs/clinical_epidemiology/oxford.asp | observational study | 1, 1.0% |
| Q-SSP (The Quality of Survey Studies in Psychology checklist) | Methodological quality | Protogerou C, Hagger M. A checklist to assess the quality of survey studies in psychology. Methods in Psychology. 2020. available at https://doi.org/10.1016/j.metip.2020.100031. | grounded theory, focus group study, semi-structured interview, questionnaire evaluation | 1, 1.0% |
| Adapted an 8-item quality assessment tool | Methodological quality | https://www.ncbi.nlm.nih.gov/pmc/articles/PMC5745693/table/tab1/ | RCT | 1, 1.0% |
| a 22-item quality assessment tool based on work by Tong, Sainsbury, and Craig (2007), Mager and Nowak (2012), and Major et al. (2018) | Reporting quality | Tong A, Sainsbury P, Craig J. Consolidated criteria for reporting qualitative research (COREQ): a 32-item checklist for interviews and focus groups. Int J Qual Health Care. 2007 Dec;19(6):349-57.  Mager U, Nowak P. Effects of student participation in decision making at school. a systematic review and synthesis of empirical research. Educational Research Review. 2012; 1(7): 38-61.  Major L, Warwick P, Rasmussen I, et al. Classroom dialogue and digital technologies: A scoping review. Educ Inf Technol. 2018 23: 1995–2028. | quantitative study, qualitative study, mixed-methods study | 1, 1.0% |
| Five criteria | / | / | / | 1, 1.0% |
| 12 criteria | / | / | RCT, crossover trial, comparative study, prospective cohort study | 1, 1.0% |
| A dichotomous nominal scale of two unique values (yes/no) | / | / | blinded cluster-randomized field trial, longitudinal study, cluster randomized 3-arm controlled trial, cluster randomized controlled trial, pre-/post-test evaluation, longitudinal quasi-experimental, pre-/-post-test study, RCT | 1, 1.0% |
| A total of 33 items indicating study quality were rated using a binary system (yes/no) | / | / | / | 1, 1.0% |
| AHRQ (Agency for Health Research Quality) | / | Sanderson S, Tatt I, Higgins J. Tools for assessing quality and susceptibility to bias in observational studies in epidemiology: a systematic review and annotated bibliography. Int J Epidemiol. 2007 Jun;36(3):666-76. | cross-sectional study | 1, 1.0% |
| Ardoin and Bowers | / | Ardoin N, Bowers A. Early childhood environmental education: A systematic review of the research literature. Educ Res Rev. 2020 Nov;31:100353. . | quantitative study, qualitative study, mixed-methods study | 1, 1.0% |
| Cochrane 5.1 | / | / | RCT, quasi-experimental study | 1, 1.0% |
| Cochranefor qualitative study quality assessment | / | Lewin S, Bohren M, Rashidian A, et al. Applying GRADE-CERQual to qualitative evidence synthesis findings-paper 2: how to make an overall CERQual assessment of confidence and create a Summary of Qualitative Findings table. Implement Sci. 2018 Jan 25;13(Suppl 1):10. | / | 1, 1.0% |
| Kirkpatrick's Hierarchy of Evaluation | / | Donald L. Evaluating Training Programs: The Four Levels. American Journal of Evaluation. 1998 August; 19(2):259-261 | cohort study | 1, 1.0% |
| Likert scale | / | Proceedings of the 19th International Conference on Evaluation and Assessment in Software Engineering, 2015, vol.27-29-April-2015, pp.14-1-14-14 | / | 1, 1.0% |
| MQS (methodological Quality Score) | / | Goodson P, Buhi E, Dunsmore S. Self-esteem and adolescent sexual behaviors, attitudes, and intentions: a systematic review. J Adolesc Health. 2006 Mar;38(3):310-9. | cross-sectional study, pre-experimental research design study, randomized cross-over design study, quasi-experimental research design study | 1, 1.0% |
| QRT (Quality Rating  Tool) | / | / | observational study | 1, 1.0% |
| RoBANs (The Risk of Bias Tool for Non-Randomized studies) | / | / | RCT, non-randomized study | 1, 1.0% |
| The QualSyst tool | / | <https://www.researchgate.net/figure/Checklist-for-assessing-the-quality-of-quantitative-studies_tbl1_284812166.>  Kmet L, Lee R, Cook L. Standard quality assessment criteria for evaluating primary research papers from a variety of fields. Edmonton: Alberta Heritage Foundation for Medical Research (AHFMR). AHFMR - HTA Initiative #13. 2004 | study with before and after design, study with retrospective before and after design, post-intervention evaluation, qualitative research | 1, 1.0% |
|  |  |  |  |  |
| Refer to Valentine and Cooper | / | Conn V, Valentine J, Cooper H, et al. Grey literature in meta-analyses. Nurs Res. 2003 Jul-Aug;52(4):256-61. | / | 1, 1.0% |
| Adapted risk-of-bias quality form of 7 items validated and adjusted for the specific context of co-educational research | / | Pfirrmann D, Herbst M, Ingelfinger P, et al. Analysis of Injury Incidences in Male Professional Adult and Elite Youth Soccer Players: A Systematic Review. J Athl Train. 2016 May;51(5):410-24. | / | 1, 1.0% |
| Adapted14 criteria adapted  from Petticrew and Roberts (2006) and Randolph (2009) | / | / | non-empirical study, empirical study | 1, 1.0% |
| Adapted 15 criteria adapted from Hirschheim (2008) and Randolph (2009) | / | / | non-empirical study, empirical study | 1, 1.0% |
| Modified Jadad rating scale | / | / | / | 1, 1.0% |
| Customized | / | / | / | 1, 1.0% |
| PRISMA (Preferred Reporting Items for Systematic reviews and Meta-Analyses)（self-created） | / | Table see <https://www.mdpi.com/2079-9292/10/14/1611/htm>  Rethlefsen M, Kirtley S, Waffenschmidt S, et al. PRISMA-S: an extension to the PRISMA Statement for Reporting Literature Searches in Systematic Reviews. Syst Rev. 2021 Jan 26;10(1):39. | / | 1, 1.0% |
| WWC (What Works Clearinghouse) Pilot Single-Case Design Standards | / | / | research with excluded group design | 1, 1.0% |
| Zhou et al | / | / | / | 1, 1.0% |
| BE2 | Evidence garding | Hinton, D. R, Robinson, M. Assessing the Strength of Evidence in the Education Sector. 2015 (p. 52). | RCT | 1, 1.0% |
| Table2 Quality Assessment Rubric | / | Brenda K. Davis. Influences on Academic Talent Development of Black Girls in K-12: A Systematic Review. Journal of Advanced Academics. 2021, 32(4), 435-468. | case study, regression, mixture modeling with auxiliary variables, phenomenological, latent class analysis, descriptive and correlational, correlational, linear mixed models, multiple regression, thematic analysis and multiple regression, hierarchical multiple regression, correlational and regression, a priori probability tests, including chi-square and correlation analysis, structural equation modeling, hierarchical cluster analysis, correlational, effect matrices, multivariate analysis, thematic content analysis, experimental crossover repeated measures design, systematic review, logistic regression, focus groups, latent variable structural equation modeling, ethnographic study, exploratory case study, multilevel modeling, ethnography | 1, 1.0% |
| QATSSD | Methodological quality | Sirriyeh, R., Lawton, R., Gardner, P., et al. Reviewing studies with diverse designs: the development and evaluation of a new tool. Journal of Evaluation in Clinical Practice. 2012, 18, 746–752. | group studies, RCT, single case experimental design studies, case series, multiple case studies | 1, 1.0% |

Note:"/" represents a lack of relevant information in the systematic reviews.
